# Effects of Notch Signaling Pathway in Cervical Cancer by Curcumin Mediated Photodynamic Therapy and Its Possible Mechanisms in Vitro and in Vivo

**DOI:** 10.7150/jca.30690

**Published:** 2019-07-10

**Authors:** Guifang He, Tianlong Mu, Yali Yuan, Wenyan Yang, Yuan Zhang, Qingyun Chen, Meilu Bian, Yanshu Pan, Qing Xiang, Zhihua Chen, Aiping Sun

**Affiliations:** 1 Department of Gynecology & Obstetrics, China-Japan Friendship Hospital, Beijing, China, 100029.; 2 Department of Pathology, Oriental Hospital of Beijing University of Chinese Medicine, Beijing, China, 100078.; 3 China-Japan Friendship School of Clinical Medicine, Peking University, Beijing, China, 100029.; 4 Department of Pathology, School of Basic Medical Sciences, Beijing University of Chinese Medicine, Beijing, China, 100029.; 5 Department of Biochemistry & Molecular Biology, Institute of Clinical Medical Science, China-Japan Friendship Hospital, Beijing , China, 100029.

**Keywords:** Notch signaling, DAPT, Photodynamic therapy, Curcumin, Cervical cancer.

## Abstract

Curcumin, as a high effect and low toxicity anti-cancer drug and photosensitiser, has synergistic and complementary effects with photodynamic therapy (PDT). However, due to its unclear mechanism, PDT's application and efficacy were limited. Notch signaling pathway, which is highly correlates with carcinogenesis and development of cervical cancer, could be a potential therapeutic targets to improve the effectiveness of PDT. Therefore, in this study, we explored the effects of Notch signaling pathway in cervical cancer by curcumin mediated PDT with/without Notch receptor blocker (DAPT), and hope to elucidate its mechanism.

Firstly, the effect on the proliferation of cervical cancer Me180 cells were detected with MTT assay, and apoptosis were detected with Annexin V-FITC/PI combined with flow cytometry. Secondly, after establishment of nude mice model, dividing the experimental animals into model group, curcumin PDT group, simple DAPT group, and curcumin-PDT+DAPT group, and analyzing tumor volume changes as well as HE staining in each group. mRNA and protein expression of gene Notch-1 and its downstream NF-κB and VEGF were observed with RT-PCR, immunohistochemical staining and Western-blot with/without inhibition of Notch signaling pathway by DAPT, both in vivo and in vitro experiments.

We found both DAPT and curcumin-PDT can inhibit the proliferation and induce apoptosis of cervical cancer cell. The two have synergistic effect in vitro and in vivo. This effect can effectively block the conduction of Notch signaling pathway, which is associated with down-regulation of the expression of Notch1 and NF-κB. Notch signaling pathway could be one of the targets of curcumin-PDT photodynamic therapy.

## Introduction

Cervical cancer is the third most common cancer in women worldwide and the fourth leading cause of tumor-induced death. China is a country with high incidence of cervical cancer in the world. About 150,000 women are infected every year, accounting for one-third of new cases globally and the incidence rate is six times higher than that of developed countries. It has become one of the most serious threat to the life and health of women in this country 1, 2. At present, the incidence of cervical cancer is moving to younger female^3^. Traditional treatment, like surgery and radio/chemo therapy, could cause serious damage to ovarian and vaginal functions^4^. While photodynamic therapy (PDT), as its high possibilities to retain patients' organ and reproductive function, is a new and effective noninvasive therapy to cancers with bright prospect. It has been proven in treatment of some malignant tumor, such as nasopharyngeal carcinoma and basal cell carcinoma^5^.

In recent years, there has been an increasing number of reports on the clinical treatment of cervical cancer in PDT. Hillemanns used ALA to treat CIN, and the remission rate of CIN was between 50%-95% 6. Choi made a bold exploratory study on the PDT treatment of early cervical cancer. 21 patients with early cervical cancer received intravenous photogem for PDT treatment. The complete remission rate was 95.2%, and only one recurred^7^.

Curcumin is a new type of photosensitizer with high efficiency, low toxicity and dual anticancer effect^8^. Most important of all, it has synergistic and complementary effects with PDT^9^. Our preliminary studies demonstrated that, PDT is an effective measure for cervical cancer treatment. However, due to its unclear mechanism, PDT's application and efficacy were limited. Notch signaling pathway, which is highly correlates with carcinogenesis and development of cervical cancer, could be a potential therapeutic targets to improve the effectiveness of PDT at the same time.

Our study is aiming to, on both cellular and molecular levels, analyze the role of Notch and downstream genes, such as NF-κB, VEGF, before and after curcumin mediated PDT on cervical cancer cell, Me180; based on which, to further observe the influences of PDT on animal model of cervical cancer; identify the possible targets in PDT on cervical cancer by blocking Notch receptor. We hope our studies could provide new ideas and experimental evidences for clinical PDT on cervical cancer.

## Materials and Methods

### Chemicals and Instruments

Curcumin, purchased from Aldrich and Sigma chemical (St. Louis, MO, USA), was dissolved in PBS, and its PH was adjusted to 7.4 by addition of 5M of NaOH. The stock solutions of 50 mmol/L were made and kept in -20^ o^ C before use. The Notch receptor blocker (N -[N- [3, 5-difluorobenzene acetyl - l-propionyl)] - (S) - phenylglycine tert-butyl, DAPT) was purchased from Calbiochem ,USA. All of the other chemicals used were of the highest purity commercially available. The 445nm laser source was purchased from Beijing Laserwave Optoelectronics Tech Co, Ltd.

### Cell culture

The Me180 cell line was purchased from Shanghai FuXiang company. The cells were routinely propagated in monolayer cultures in Dulbecco's modified Eagles' medium (Gibco-BRL, Paisley, Scotland), supplemented with 5% heat-inactivated fetal bovine serum, 0.37% sodium bicarbonate, 30mM HEPES, and penicillin/streptomycin. The cells were cultured in a 5% CO_2_ incubator at 37^ o^ C. The cells were serially subcultured normally and the cells at the logarithmic growth phase were chosen for experiments.

### Cell proliferation assay

The Me180 cell lines were inoculated into an 96-well at a volume of 100μl (5×10^4^cells/well) for stationary culture. Our preliminary orthogonal experimental research shows that curcumin mediated PDT induced apoptosis of cervical cancer cells with the best combination of curcumin concentration of 5μmol/L, laser dose of 100J /cm^2^, irradiation time of 180s, incubation time of 24h. Adding different concentrations of DAPT 2 hours before PDT. The treatment factors were added and divided into control group, simple DAPT group, curcumin-PDT group and curcumin-PDT+DAPT group for routine MTT detection. MTT method was used to calculate interaction index (CDI) of the two drugs, including DAPT and curcumin-PDT. The synergistic effect of DAPT and curcumin-PDT was judged by combined index (CI). The formula is CI=AB / (A×B). A and B represent two different drugs respectively. AB was the T/C value of the combination of the two drugs, A and B were the T/C value of the single drug, and T/C value was the ratio of the drug group to the control group.

### Flow cytometry

Me180 cells were seeded in 6 Hole culture plates and cultured for 24 h, then culture medium replaced. At this time, DAPT (1μmol/L) and curcumin (2.5μmol/L) were added and blank control was set up. After incubation for 6 hours, fresh culture medium was changed immediately after 180 seconds irradiated with 100 J/cm ^2^ laser. After 24 hours of continuous culture, the cells of four groups were collected and cleaned by PBS twice. Cell suspensions were centrifuged and resuspended in PBS to a concentration of 10^6^ cells/ml. For flow cytometric analysis, cells were incubated with 5 μl of Annexin V-fluorescein isothiocyanate (Beijing baosai biotech co, ltd.) and 10 μl of propidium iodide (Sigma chemical, USA) in the dark at room temperature for 10 min followed by fixation with 2% formaldehyde. The stained cells were analyzed for DNA content by fluorescence activated cell sorting (FACS) in a FACScan (Beckman Coulter Epics XL, USA). The forward- and side-scatter gates were set to exclude any dead cells from the analysis. At least 10,000 events were collected for each sample.

### Real-time Reverse Transcription-PCR

Total mRNA was isolated from Me180 cell lines of 4 groups and cells using Trizol Reagent (QIAGEN's RNeasy® RNA extraction kit) according to manufacturer's instructions. After DNAse I treatment, the RNA was reverse transcribed and the cDNA was used for real-time PCR. Real-time PCR was performed with the following sets of primers:

β-actin: forward: 5'-TTGTTACAGGAAGTCCCTTGCC-3'; reverse: 5'-ATGCTATCACCTCCCCTGTGTG-3'.

Notch1: forward: 5'-CGGAGTGTGTATGCCAAGAGT-3'; reverse: 5'-GGTTCTGGAGGGACCAAGA-3'.

2^-ΔCt^ method was used to calculate the relative mRNA level of each group. The ΔCt values was calculated according to the formula: ΔCt_target gene of PDT group_ = Ct _target gene of PDT group_ - Ct_ β-actin of the same sample_ ;ΔCt _target gene of control group_ = Ct _target gene of control group_ - Ct_β-actin of the same sample_; Values of ΔCt between different groups were compared.

### Western blot

Me180 cells of 4 groups were plated in 6-well plates at the density of 1×10^6^ cells per well in 2 mL of theculture medium and cultured at 37^ o^C overnight under 5% CO_2_. Cells were collected and lysed after 60 minutes, and total protein concentrations were determined with a Bio-Rad BCA® kit. Equal amounts of cell lysates were loaded onto 10% SDS gel and separated by electrophoresis. Separated proteins were then electro-transferred onto polyvinylidene fluoride (PVDF) membranes (Millipore, Bedford, MA). After being blocked with 1× Tris-buffered saline (TBS) containing 0.1% Tween-20 and 5% bovine serum albumin (BSA), the membranes were incubated with primary antibodies at room temperature for 2 hours or at 4^ o^C O/N, then washed with 1×TBS containing 0.1% Tween-20 and followed by treatment with the horseradish peroxidase (HRP)-conjugated secondary antibody at room temperature for another 1 hour. The targeted proteins were visualized using an enhanced chemiluminescence (ECL) plus system (Thermo Fisher Scientific, Waltham, MA). Primary antibodies against Notch1 were purchased from Abcam (Cambridge, UK).NF-κB, VEGF were purchased from Santa Cruz Biotechnology. β-Actin and the horseradish peroxidase (HRP) conjugated goat anti-rabbit IgG were purchased from Cell Signaling Technology.

### Animals

Female BALB/c nude mice were obtained from Vitalriver Laboratory Animal Technology Co. (Beijing, China) and maintained under specific pathogen-free conditions. The mice were 6-8 weeks old and weighed 20-25 g when the experiments started.

### Animal model

About 5×10^6^ cells ( 200 μL) were injected subcutaneously into the back of BALB/C nude mice to set up cervical cancer xenograft model. After successful establishment of the model, experimental animals are divided into four groups with 12 animals each (model group, the curcumin-PDT group, only DAPT group and curcumin-PDT with DAPT group).

(A) In the model group, 200μL saline was injected around the tumor and then natural light was given. (B) In curcumin mediated PDT group without Notch blocker, curcumin solution of 150μL was injected locally around the tumor for 200μL, and PDT was performed 4 hours later at a dose of 100 J/cm^2^. (C) In the single DAPT group, DAPT solution was injected intraperitoneally every other day from the first week before treatment with a dose of 100 mg/kg. (D) In curcumin-PDT therapy combined with Notch blocker group, One week before PDT, DAPT solution was injected intraperitoneally every other day from the first week before treatment with a dose of 100 mg/kg. Then on the day of treatment, curcumin solution of 150μL was injected locally around the tumor for 200μL. PDT was performed 4 hours later at a dose of 100 J/cm^2^.

Each group has 12 mice. After one-day treatment, 6 mice were killed to cut tumor tissue for follow-up experiments. The remaining 6 mice continued to record tumor volume and tumor size. And it was measured 2 times per week until 21 days after treatment.

### Inhibition of tumor growth

The tumor volume was calculated and tumor growth curve was drawn. Tumor volume V=a×b^2^/2 (a is the longest diameter of tumor and b is the shortest ). Observe and record any adverse reactions that occur during the treatment, including weight loss, death, etc.

### Histopathologic examination

After measuring the gross tumor size, the specimens were embedded in paraffin, cut into 5-μm slices, and mounted on slides. The tumor xenografts was stained with hematoxylin-eosin and examined under a light microscope.

### Immunohistochemical staining

After fixed in 4% paraformaldehyde for 1 day, the tumor was routinely dehydrated and embedded to prepare the pathological sections, which were stained to observe the changes of the expression of Notch, NF-κB, and VEGF in each group. Briefly, tissue sections were incubated overnight at 4°C with an anti- Notch1(Abcam Cambridge, UK), NF-κB, and VEGF monoclonal antibody (Santa Cruz Biotechnology). Then, the sections were incubated 40 min with biotinylated anti-rabbit secondary antibody, and another 40 min with the avidin-biotinylated peroxidase complex. The sections were washed with distilled water (10 min), treated with diaminobenzidine (DAB) to visualize positive staining. IPP6.0 analysis software was used for image analysis to measure optical density (IOD) value. Based on the average of the model group, the percentage of the data divided by the model group is used to analyze the immunohistochemical results.

### Statistical analysis

The experiments were carried out in triplicate and values were shown as the mean± standard deviation (SD). The single factor variance analysis was used between groups. All statistical tests with P<0.05 were considered significant. SPSS 19.0 software was used for statistical analysis.

## Results

### Effects of curcumin-PDT and DAPT on the survival rate of cervical cancer Me180 cells

**Figure 1:**
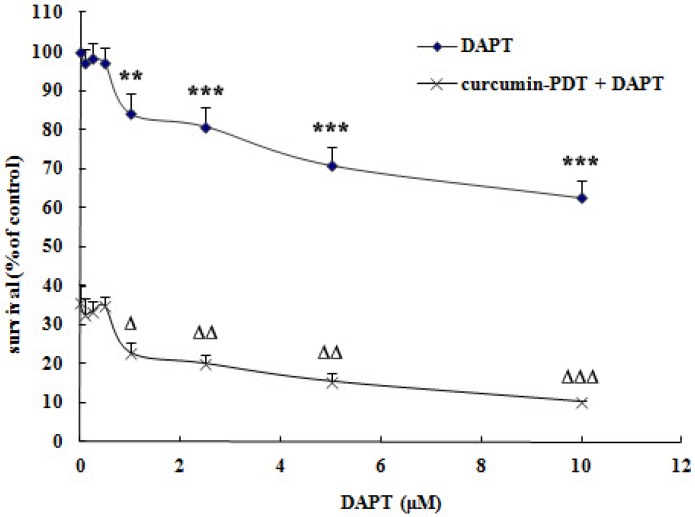
MTT assay. Effect of combination of different concentrations of DAPT and curcumin photodynamic therapy on survival rate of cervical cancer Me180 cells. Compared with the control group, the difference was significant (*P<0.05, ** P<0.01, *** P<0.001; ^∆^ P<0.05, ^∆∆^ P<0.01,^ ∆∆∆^ P<0.001). Curcumin -PDT and DAPT have moderate synergistic effects.

DAPT itself can inhibit the growth of cervical cancer Me180 cells. When the concentration of DAPT was 1μmol/L or above, it could significantly inhibit the growth of Me180 cells. After curcumin-PDT treatment, the Notch signaling pathway was blocked by DAPT, and the inhibition of cell proliferation was more significant (Fig. 1). The inhibition rate of 1μmol/L DAPT on Me180 cells was 15.79%. However, that of curcumin -PDT combined with blocker DAPT was 77.18%. As shown in Table 1, when curcumin -PDT was added to the DAPT group, the concentration of DAPT was greater than 1 μmol/L and CI<0.8. Both have moderate synergistic effects.

### Curcumin -PDT and DAPT induces apoptosis in cervical cancer Me180 cells

**Figure 2:**
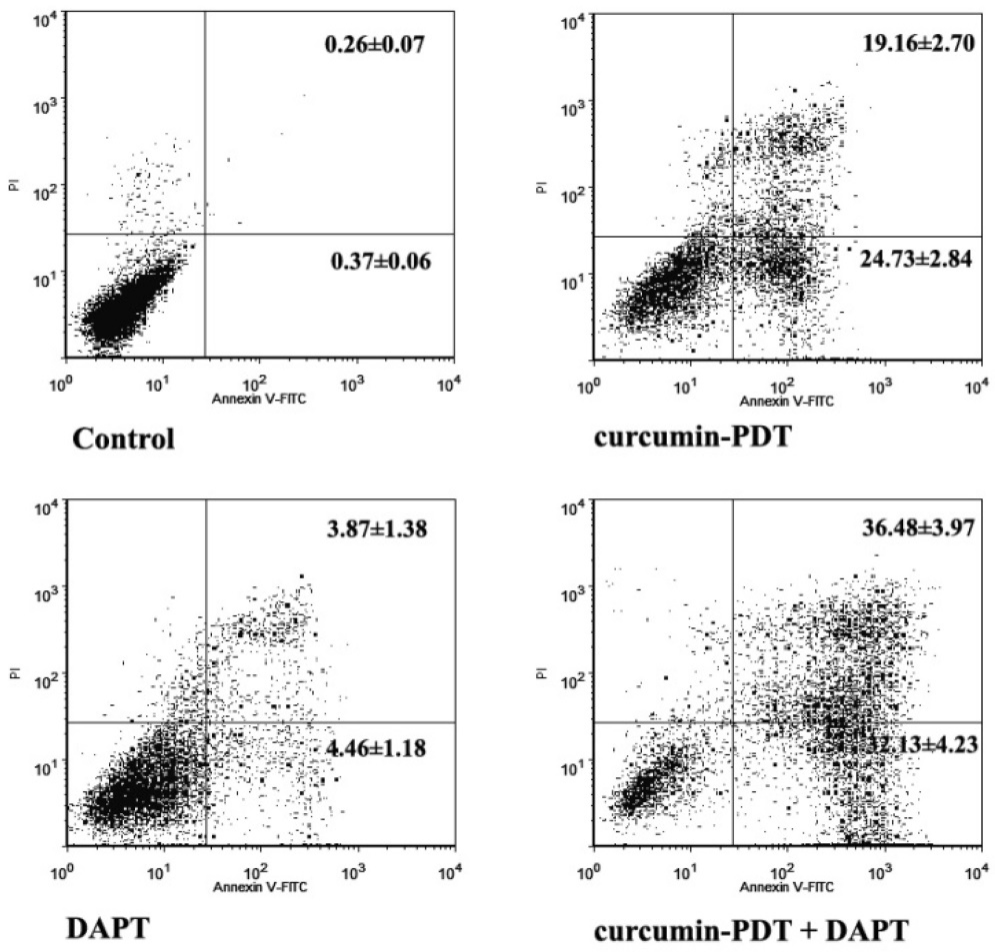
Cell apoptosis analysis by flow cytometry of curcumin-PDT and DAPT in Me180 cells stained with Annexin-V and PI.

Both curcumin-PDT and 1μmol/L DAPT can induce apoptosis in cervical cancer cell line Me180. The total mortality rate of the former is 43.9% and the latter is 8.33%. There were significant differences between early and late apoptosis rates. After curcumin-PDT plus DAPT, there was a synergistic interaction between curcumin-PDT and DAPT, and the difference was significant(P<0.01). The total mortality rate is 68.61% (Fig. 2).

### Downregulation of Notch1 mRNA expression in vitro

Curcumin-PDT and DAPT both inhibited Notch1 mRNA expression in Me180 cervical cancer cells. The inhibition rates were 32.33% and 39.99% respectively (Table 2). Curcumin-PDT combined with DAPT could inhibit Notch1 most significantly. Curcumin-PDT combined with DAPT had synergistic effects. The inhibition rate was 79.27%. Compared with the control group and curcumin-PDT, the differences between them were statistically significant (P < 0.05 or P < 0.01).

### Protein expression of Notch and downstream genes detected by western blot in vitro

**Figure 3:**
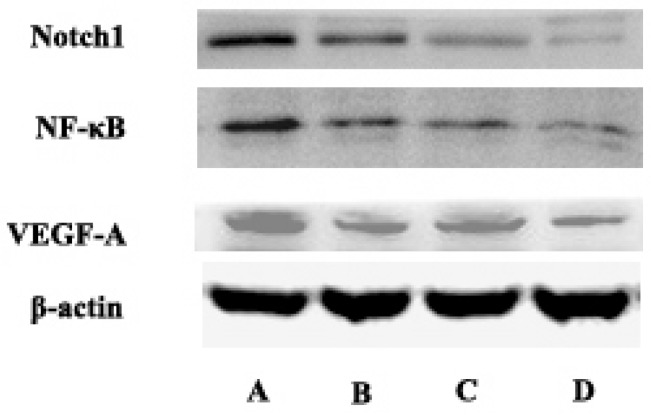
The express of Notch1, NF-κB and VEGF-A in A (control group), B (Curcumin-PDT group), C (DAPT group) and D (Curcumin-PDT combined with DAPT group) cells by Western blot. The results showed that the expression of Notch1, NF-κB and VEGF-A downregulated significantly in the Curcumin-PDT combined with DAPT group compared with other groups.

Compared with the control group, curcumin-PDT group significantly inhibited the expression of Notch1, NF-κB and VEGF-A. Compared with the control group, DAPT also inhibited the expression of Notch1, NF-κB and VEGF-A. When curcumin-PDT was blocked by DAPT, the expression of Notch1, NF-κB and VEGF-C was inhibited more significantly than that of the two alone (Fig. 3).

### Xenograft tumor growth inhibition by curcumin-PDT

**Figure 4:**
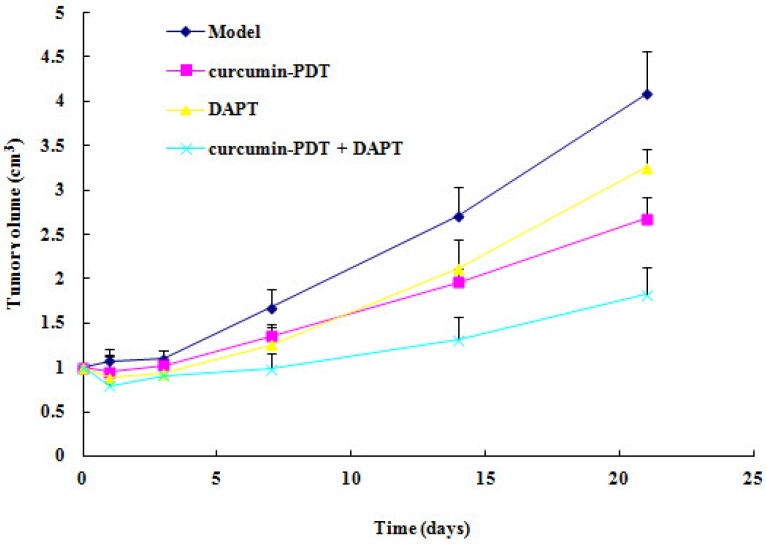
The xenograft tumor growth curve. The results showed curcumin-PDT suppressed the tumor growth (n=6, P < 0.05).

We used Me180 cell xenograft nude mice to investigate PDT anticancer activity in vivo. Tumor volume in model group increased gradually. Tumor volume of curcumin-PDT group, DAPT group, curcumin-PDT+DAPT group remained unchanged or slightly decreased from 1 to 7 days after treatment (Fig. 4). There was significantly different from the model group (P < 0.05). Then they were gradually increased after 14 days.

### Pathological morphology observation of xenografts after curcumin-PDT

**Figure 5:**
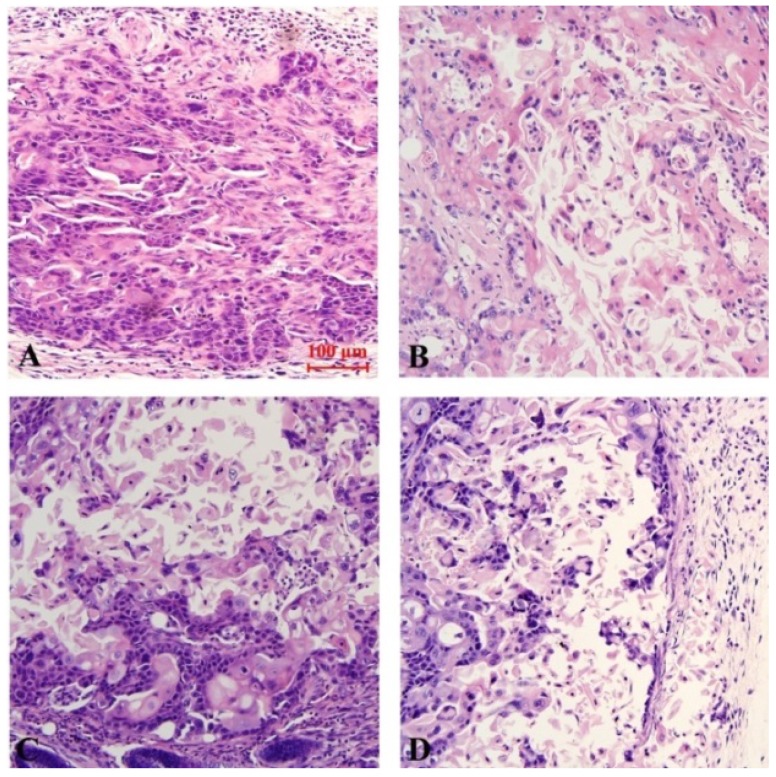
Histopathological staining of Xenograft tissue in nude mice (HE staining, x 200). A (control group), B (Curcumin-PDT group), C (DAPT group) and D (Curcumin-PDT combined with DAPT group).

The results of Hematoxylin-Eosin (HE) staining demonstrate that, there is no appreciable change in the control group. The tumor cells were nested in the model group. Cell components are relatively single and relatively large in size. The cell boundaries were not clear, ranging from round to oval. Chromatin is rough and mitotic figures are common. Occasionally necrosis occurs. However, compared with the model group, the cells in each treatment group arranged sparsely. The volume was reduced, the ratio of nucleus to plasma was reduced, part of the nucleus was pyknosis, vesicular nucleus and perinuclear halo were observed, and the mitotic image was less than the model group. Among them, the necrosis of curcumin-PDT with blocker DAPT was the most obvious (Fig. 5).

### Downregulation of Notch1 mRNA expression in vivo

Curcumin-PDT and DAPT both inhibited Notch1 mRNA expression in cervical cancer xenografts. The inhibition rates were 40.54% and 42.17% respectively (Table 3). Curcumin-PDT combined with DAPT could inhibit Notch1 most significantly. Curcumin-PDT combined with DAPT had synergistic effects. The inhibition rate was 79.22%. Compared with the control group and curcumin-PDT, the differences between them were statistically significant (P < 0.05 or P < 0.01).

### Immunohistochemical assessment and Western Blot analysis of the protein expression of Notch and its downstream genes in vivo

**Figure 6:**
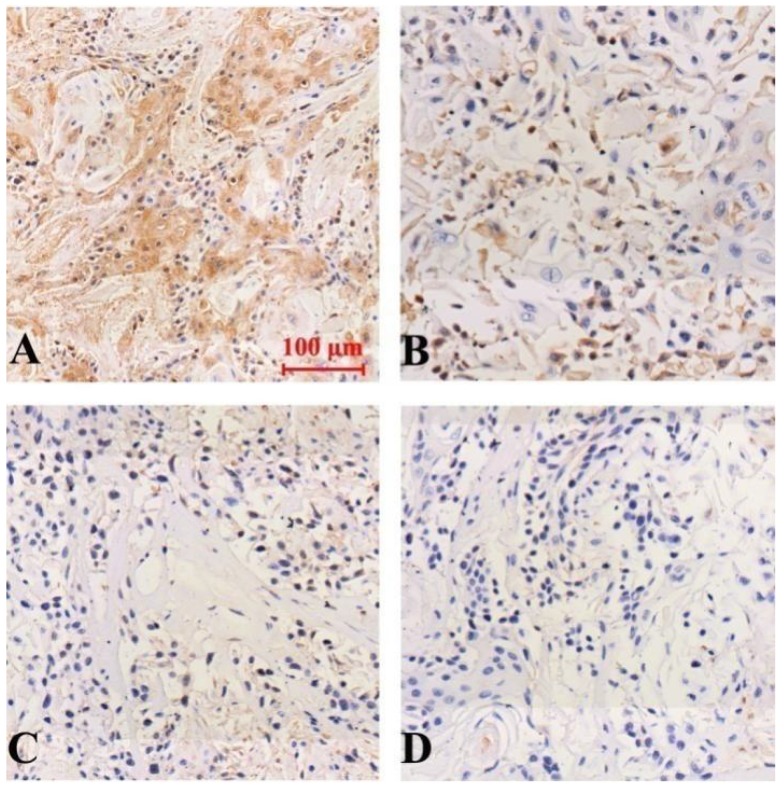
Expression of Notch-1 protein in tumor-bearing nude mice tumor tissue (Immunohistochemical staining, x 200). A (model group), B (curcumin-PDT group), C (DAPT group), D (curcumin-PDT + DAPT group).

**Figure 7:**
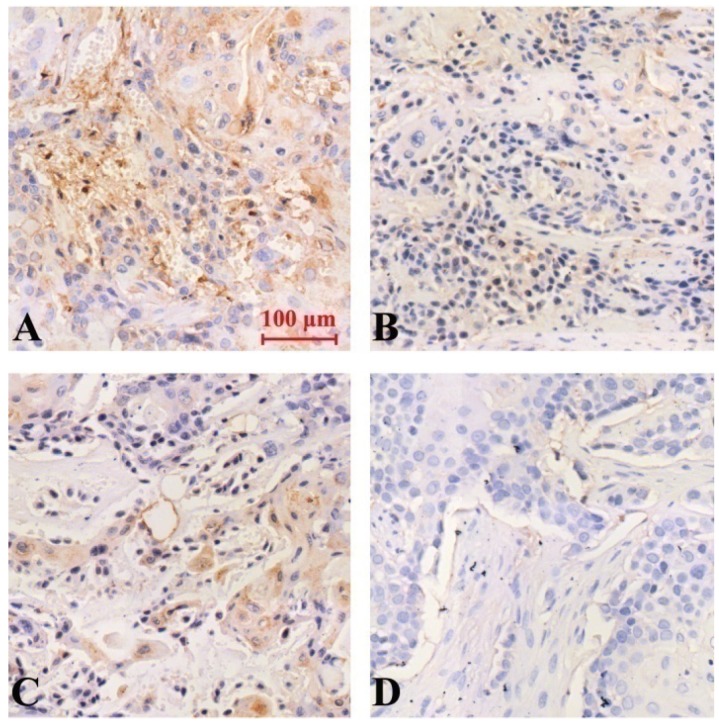
Expression of NF-κB protein in tumor-bearing nude mice tumor tissue (Immunohistochemical staining, x 200). A (model group), B (curcumin-PDT group), C (DAPT group), D (curcumin-PDT + DAPT group).

**Figure 8:**
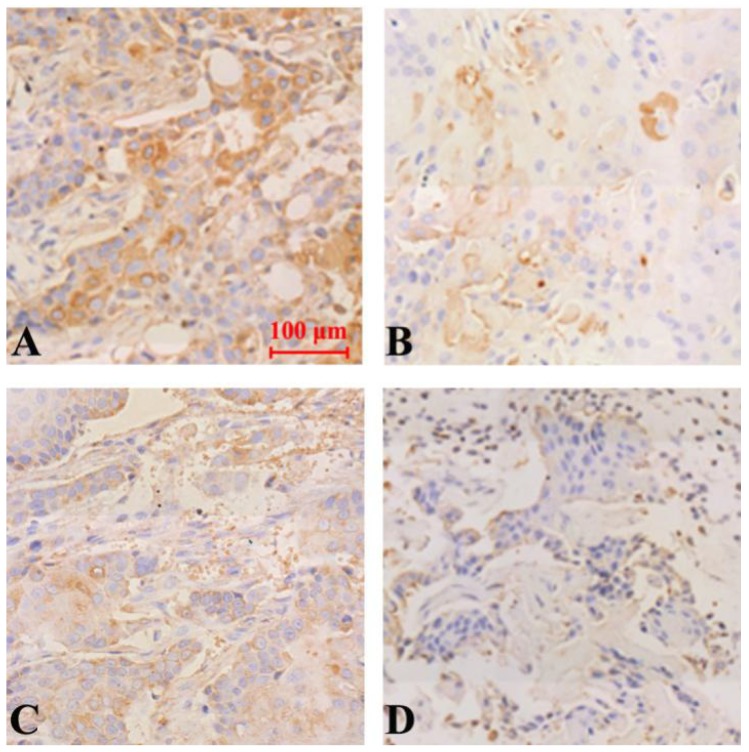
Expression of VEGF protein in tumor-bearing nude mice tumor tissue (Immunohistochemical staining, x 200). A (model group), B (curcumin-PDT group), C (DAPT group), D (curcumin-PDT + DAPT group).

The results of immunohistochemical staining showed that the expression of Notch-1, NF-κB and VEGF protein was down-regulated in all groups compared with the model group, and the difference was statistically significant (P<0.05). Curcumin-PDT with DAPT group had the strongest inhibitory effect Compared with curcumin-PDT group and DAPT group, the difference was statistically significant (P < 0.05). The inhibitory effect of DAPT on Notch-1 expression was slightly stronger than that of curcumin-PDT (P < 0.05) (Fig. 6), but the inhibitory effect on the expression of NF-κB (Fig. 7) and VEGF-A (Fig. 8) was slightly weaker than that of curcumin-PDT (P < 0.05).

**Figure 9:**
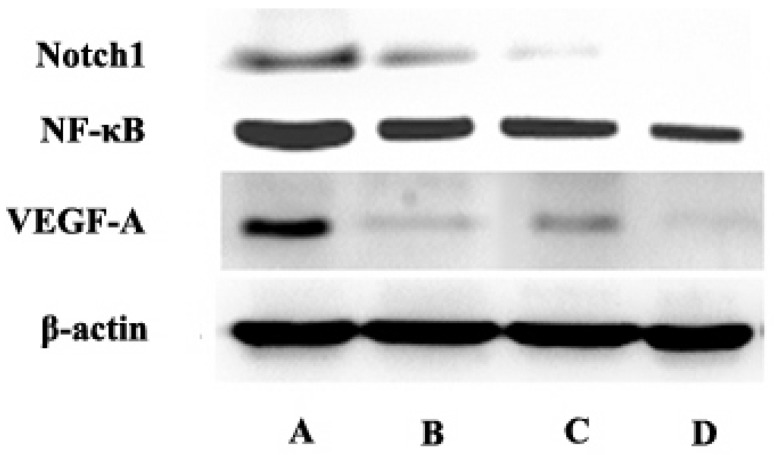
The express of Notch1, NF-κB and VEGF-A in A (control group), B (Curcumin-PDT group), C (DAPT group) and D (Curcumin-PDT combined with DAPT group) tumor tissues by Western blot. The results showed that the expression of Notch1, NF-κB and VEGF-A down-regulated significantly in the D group compared with other groups.

For Notch and its downstream genes and protein expression downregulation, it was verified by western blot further. There was no significant difference in the expression of NF-κB and VEGF-A between curcumin-PDT and DAPT alone group. In addition to the above differences, the remaining results were consistent with immunohistochemistry (Fig. 9).

## Discussion

Cervical cancer is the second leading cause of death in women with gynecological malignancies^10^, ^11^. There are nearly 500,000 new cases and about 270,000 deaths worldwide every year 12. In some developing countries, the incidence rate of cervical cancer ranks first 13. However, a large number of studies and practices have shown that cervical cancer is the only type of malignant tumor whose morbidity and mortality can be reduced by medical intervention 14.

With the development of Nano-tech, Biotech and Photology, and their fusion with Medical Science, photodynamic therapy become an increasingly valid alternative for cervical cancer treatment. It can treat early tumors and also effective in the treatment of advanced tumors 15. Photodynamic therapy (PDT) is an FDA-approved anticancer modality that has been shown to enhance anti-tumor immunity. Numerous studies have shown that stimulation of the host immune system can result in the generation of anti-tumor immune responses capable of controlling metastatic tumor growth 16. Studies have reported that refractory advanced lung cancer can be alleviated by photodynamic therapy (PDT) combined with chemotherapy 17. Photodynamic therapy has achieved certain curative effect. This study confirmed the effectiveness of PDT in the treatment of cervical cancer in vitro and in vivo, which echos the results of previous reports done by other researchers.

Mogan, a genetic pioneer, first discovered Notch signaling protein in 1916, named it because a partially disabled mutant would create a notch at the edge of a fruit fly's wing 18. A total of four Notch genes, 9q34, 1p13-p11, 19p13.2-p13.1 and 6p21.3, were identified in mammals, encoding four Notch receptors (Notch1-4) 19. However, the relationship between Notch and tumor is complex, not only promoting cancer but also inhibiting cancer in different circumstances 20. It is reported that Notch is highly expressed in low-grade cervical tumors. In a more aggressive, high-grade cervical cancer, the expression is lower 21. Daniel et al. found that the expression of activated Notch-1 was closely related to the severity of HPV-related early cervical epithelial injury 22. Notch-1 signaling pathway is involved in the occurrence and development of cervical cancer 23. Clinical impact of de-regulated Notch-1 and Notch-3 in the development and progression of HPV-associated different histological subtypes of precancerous and cancerous lesions of human uterine cervix was studied. The findings suggest that Notch-1 and Notch-3 may play an important role with synergistic effect of HPV in regulating development and proliferation of cervical cancer through the deregulation of Notch signalling 24. The abnormal expression of Notch signaling pathway is related to the occurrence and progression of cervical cancer 25. Therefore, to explore the mechanism of Notch in PDT treatment of cervical cancer and to fully understand the regulation of its downstream genes by Notch will help enriching the strategy of PDT treatment and expand its clinical application.

We studied the effects and mechanisms of Notch signaling pathway in cervical cancer by curcumin mediated PDT. In addition, we identify the possible targets in PDT on cervical cancer through blocking Notch receptor. γ-secretase inhibitor (DAPT) was chosen to block Notch signaling pathway. Our studies had shown that DAPT itself inhibited Notch-1 mRNA and protein expression in cervical cancer in vitro and in vivo. DAPT and curcumin PDT demostrates great synergistic effects. After curcumin-PDT was added into the blocking agent DAPT, the efficacy of PDT could be further improved. Activated expression of Notch-1 protein may be associated with carcinogenesis of normal cervical epithelium and may affect the development of cervical cancer. Therefore, we have good reasons to suspect that Notch-1 is one of the key targets for curcumin-PDT in the treatment of cervical cancer. It is suggested that the therapeutic effect of PDT could be improved by regulating the target of Notch-1 in future clinical practice.

The regulation of cell differentiation and apoptosis by Notch signaling pathway is very complex 26. PDT treatment on signaling pathways with different photosensitizers and on different cell types also lead to different results 27. Moreover, it increases the complexity of its mechanism of action, and generates various efficacy of PDT in different diseases. Based on the results of our studies, Notch signaling pathway is one of the key, however not the only, targets among the mechanism of PDT. We analyzed and concluded the complexity and diversity of the mechanism could be related to the following factors: (1) Notch itself is divided into four types. Although the members of Notch pathway are fixed, the ligands and target genes of Notch pathway have different subtypes, so their roles in different tumors are different 28. (2) There are interactions between Notch and other signaling pathways, such as Ras and Wnt, which can also regulate cell's growth by activating Notch pathway 29. (3) Notch is ubiquitous in many tissues and cells, and is highly conservative in biological evolution 30. Many different stimuli can activate Notch pathway. Sharing of Notch signaling pathway is one of the reasons for its complex function.

Notch pathway controls the activation of NF-κB pathway by activating CSL protein in the side effector cells 31. An important downstream target gene of NF-κB pathway is VEGF, which is closely related to the invasion and metastasis of cervical cancer^32^. In this study, our observation proved that curcumin-PDT can block Notch signaling pathway and tune down the expression of key factors such as Notch-1 and NF-κB, thus inhibiting tumor growth.

In conclusion, this study successfully demonstrated that Curcumin-PDT combined with DAPT could inhibit the expression of Notch-1 and its downstream related proteins in cervical cancer in vitro and in vivo. We conclude that Notch-1 could be one of the targets of curcumin-PDT in the treatment of cervical cancer. Receptor blocker DAPT has synergistic effect on curcumin-PDT in the treatment of cervical cancer, which is mainly related to the down-regulation of Notch-1 and NF- κB expression.

## Figures and Tables

**Table 1:** Inhibition of sigle DAPT and combined with curcumin -PDT and coefficient of drug interaction(CDI). (^*^P<0.05,^ **^ P<0.01, ^***^ P<0.001;^∆^ P<0.05,^∆∆^ P<0.01, ^∆∆∆^ P<0.001)

DAPT (μM)	DAPT	curcumin-PDT+DAPT	CDI
0	100.000	35.43±4.62^***^	1.00
0.10	97.25±3.38	32.62±4.01	0.95
0.25	98.13±4.23	33.16±2.97	0.95
0.50	97.11±4.07	34.71±2.32	1.01
1.00	84.21±4.84^**^	22.82±2.72^∆^	0.76
2.50	80.83±4.80^***^	19.98±2.32^∆∆^	0.70
5.00	70.89±4.81^***^	15.45±2.23^∆∆^	0.61
10.00	62.54±4.25^***^	10.20±0.49^∆∆∆^	0.46

**Table 2:** The expression of Notch1 mRNA expression in Me180 cervical cancer cells. Curcumin-PDT combined with DAPT could inhibit Notch1 most significantly compared with other groups. (Comparison with Control group, ^*^ P< 0.05, ^**^ P< 0.01,^ ***^ P< 0.001; Compared with the curcumin -PDT group, ^∆^ P< 0.05,^ ∆∆^ P< 0.01, ^∆∆∆^P< 0.001; Compared with the DAPT group, it was ^●^P< 0.05, and ^●●^P< 0.01, ^●●●^P< 0.001).

group	β-actin	Notch1	△Ct	Inhibition rate
Control	21.17±0.08	23.10±0.06	1.93±0.04	-
Curcumin-PDT	21.20±0.09	23.69±0.13	2.49±0.20^*^	32.33%
DAPT(1 μM)	21.39±0.21	24.05±0.09	2.67±0.16^**^	39.99%
curcumin-PDT+DAPT	21.15±0.28	25.35±0.24	4.20±0.35^***∆∆∆●●●^	79.27%

**Table 3:** The inhibition rate of Notch1 mRNA in cervical cancer xenografts (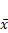 ± *s*, n = 3) Compared with the model group, ^*^ P <0.05, ^**^ P <0.01, ^***^ P <0.001; Compared with curcumin-PDT group, ^Δ^P <0.05, ^ΔΔ^P <0.01, ^ΔΔΔ^P <0.001; Compared with DAPT group, ^●^ P <0.05, ^●●^ P <0.01, ^●●●^ P <0.001.

Groups	β-actin	Notch1	△Ct	Inhibition rate
model group	22.46±0.11	24.29±0.27	1.84±0.32	-
curcumin-PDT	22.55±0.29	25.14±0.20	2.59±0.09*	40.54%
DAPT	22.55±0.20	25.18±0.11	2.63±0.21**	42.17%
curcumin-PDT+DAPT	22.88±0.25	26.98±0.14	4.10±0.23***^∆∆∆●●●^	79.22%

## References

[1] Shrestha AD, Neupane D, Vedsted P (2018). Cervical Cancer Prevalence, Incidence and Mortality in Low and Middle Income Countries: A Systematic Review. Asian Pac J Cancer Prev.

[2] Wu ES, Jeronimo J, Feldman S (2017). Barriers and challenges to treatment alternatives for early-Stage cervical cancer in lower-resource settings. J Glob Oncol.

[3] Kessler TA (2017). Cervical Cancer: Prevention and Early Detection. Semin Oncol Nurs.

[4] LaVigne AW, Triedman SA, Randall TC (2017). Cervical cancer in low and middle income countries: Addressing barriers to radiotherapy delivery. Gynecol Oncol Rep.

[5] Kwiatkowski S, Knap B, Przystupski D (2018). Photodynamic therapy-mechanisms, photosensitizers and combinations. Biomed Pharmacother.

[6] Hillemanns P, Garcia F, Petry KU (2015). A randomized study of hexaminolevulinate photodynamic therapy in patients with cervical intraepithelial neoplasia 1/2. Am J Obstet Gynecol.

[7] Choi MC, Jung SG, Park H (2014). Fertility preservation by photodynamic therapy combined with conization in young patients with early stage cervical cancer: a pilot study. Photodiagnosis Photodyn Ther.

[8] Abrahamse H, Hamblin MR (2016). New photosensitizers for photodynamic therapy. Biochem J.

[9] de Matos RPA, Calmon MF, Amantino CF (2018). Effect of Curcumin-Nanoemulsion Associated with Photodynamic Therapy in Cervical Carcinoma Cell Lines. Biomed Res Int.

[10] Landoni F, Colombo A, Milani R (2017). Randomized study between radical surgery and radiotherapy for the treatment of stage IB-IIA cervical cancer: 20-year update. J Gynecol Oncol.

[11] Small W Jr, Bacon MA, Bajaj A (2017). Cervical cancer: A global health crisis. Cancer.

[12] Daniyal M, Akhtar N, Ahmad S (2015). Update knowledge on cervical cancer incidence and prevalence in Asia. Asian Pac J Cancer Prev.

[13] Kumar L, Harish P, Malik PS (2018). Chemotherapy and targeted therapy in the management of cervical cancer. Curr Probl Cancer.

[14] Bogani G, Leone Roberti Maggiore U, Signorelli M (2018). The role of human papillomavirus vaccines in cervical cancer: Prevention and treatment. Crit Rev Oncol Hematol.

[15] Fadel M, Kassab K, Abd El Fadeel DA (2018). Comparative enhancement of curcumin cytotoxic photodynamic activity by nanoliposomes and gold nanoparticles with pharmacological appraisal in HepG2 cancer cells and Erlich solid tumor model. Drug Dev Ind Pharm.

[16] Shams M, Owczarczak B, Manderscheid-Kern P (2015). Development of photodynamic therapy regimens that control primary tumor growth and inhibit secondary disease. Cancer Immunol Immunother.

[17] Kimura M, Miyajima K, Kojika M (2015). Photodynamic Therapy (PDT) with Chemotherapy for Advanced Lung Cancer with Airway Stenosis. Int J Mol Sci.

[18] Dueñas-González A, Campbell S (2016). Global strategies for the treatment of early-stage and advanced cervical cancer. Curr Opin Obstet Gynecol.

[19] Capaccione KM, Pine SR (2013). The Notch signaling pathway as a mediator of tumor survival. Carcinogenesis.

[20] Aster JC, Pear WS, Blacklow SC (2017). The varied roles of notch in cancer. Annu Rev Pathol.

[21] Artavanis-Tsakonas S, Rand MD, Lake RJ (1999). Notch-1 signaling: cell fate control and signal integration in development. J Science.

[22] Daniel B, Rangarajan A, Mukherjee G (1997). The link between integration and expression of human papillomavirus type16 genomes and cellular changes in the evolution of cervical intraepithelial neoplastic lesions. J Gen Virol.

[23] Maliekal TT, Bajaj J, Giri V (2008). The role of Notch signaling in human cervical cancer: implications for solid tumors. Oncogene.

[24] Tripathi R, Rath G, Jawanjal P (2014). Clinical impact of de-regulated Notch-1 and Notch-3 in the development and progression of HPV-associated different histological subtypes of precancerous and cancerous lesions of human uterine cervix. PLoS One.

[25] Zagouras P, Stifani S, Blaumueller CM (1995). Alteraction in Notch signaling in neoplastic lesions of the human cervix. Proc Natl Acad Sci USA.

[26] Dang TP (2012). Notch, apoptosis and cancer. Adv Exp Med Biol.

[27] Nwabo Kamdje AH, Takam Kamga P, Tagne Simo R (2017). Developmental pathways associated with cancer metastasis: Notch, Wnt, and Hedgehog. Cancer Biol Med.

[28] Majidinia M, Alizadeh E, Yousefi B (2016). Downregulation of Notch signaling pathway as an effective chemosensitizer for cancer treatment. Drug Res.

[29] Oh SJ, Ahn S, Jin YH (2015). Notch 1 and Notch 2 synergistically regulate the differentiation and function of invariant NKT cells. J Leukoc Biol.

[30] Heck BW, Zhang B, Tong X (2012). The transcriptional corepressor SMRTER influences both Notch and ecdysone signaling during Drosophila development. Biol Open.

[31] Kumar V, Palermo R, Talora C (2014). Notch and NF-kB signaling pathways regulate miR-223/FBXW7 axis in T-cell acute lymphoblastic leukemia. Leukemia.

[32] Spirina LV, Usynin YA, Yurmazov ZA (2017). Transcription factors NF-kB, HIF-1, HIF-2, growth factor VEGF, VEGFR2 and carboanhydrase IX mRNA and protein level in the development of kidney cancer metastasis. Mol Biol.

